# Organosolv pretreatment: an in-depth purview of mechanics of the system

**DOI:** 10.1186/s40643-023-00673-0

**Published:** 2023-08-11

**Authors:** Lakshana G. Nair, Komal Agrawal, Pradeep Verma

**Affiliations:** 1 Bioprocess and Bioenergy Laboratory, Department of Microbiology, Central University of Rajasthan, Bandarsindri, Kishangarh, Ajmer, Rajasthan 305817 India; 2 Department of Microbiology, School of Bio Engineering and Biosciences, Lovely Professional University, Phagwara, Punjab 144411 India

**Keywords:** Lignocellulose biomass, Organic solvent, Pretreatment, Lignin, Cellulose

## Abstract

The concept of biorefinery has been advancing globally and organosolv pretreatment strategy has seen an upsurge in research due to its efficiency in removing the recalcitrant lignin and dissolution of cellulose. The high-performance organosolv system uses green solvents and its reusability contributes concurrently to the biorefinery sector and sustainability. The major advantage of the current system involves the continuous removal of lignin to enhance cellulose accessibility, thereby easing the later biorefinery steps, which were immensely restricted due to the recalcitrant lignin. The current system process can be further explored and enhanced via the amalgamation of new technologies, which is still a work in progress. Thus, the current review summarizes organosolv pretreatment and the range of solvents used, along with a detailed mechanistic approach that results in efficient pretreatment of LCB. The latest developments for designing high-performance pretreatment systems, their pitfalls, and advanced assessments such as Life Cycle Assessment along with Techno-Economic Assessment have also been deliberated to allow an insight into its diverse potential applicability towards a sustainable future.

**Graphical Abstract:**

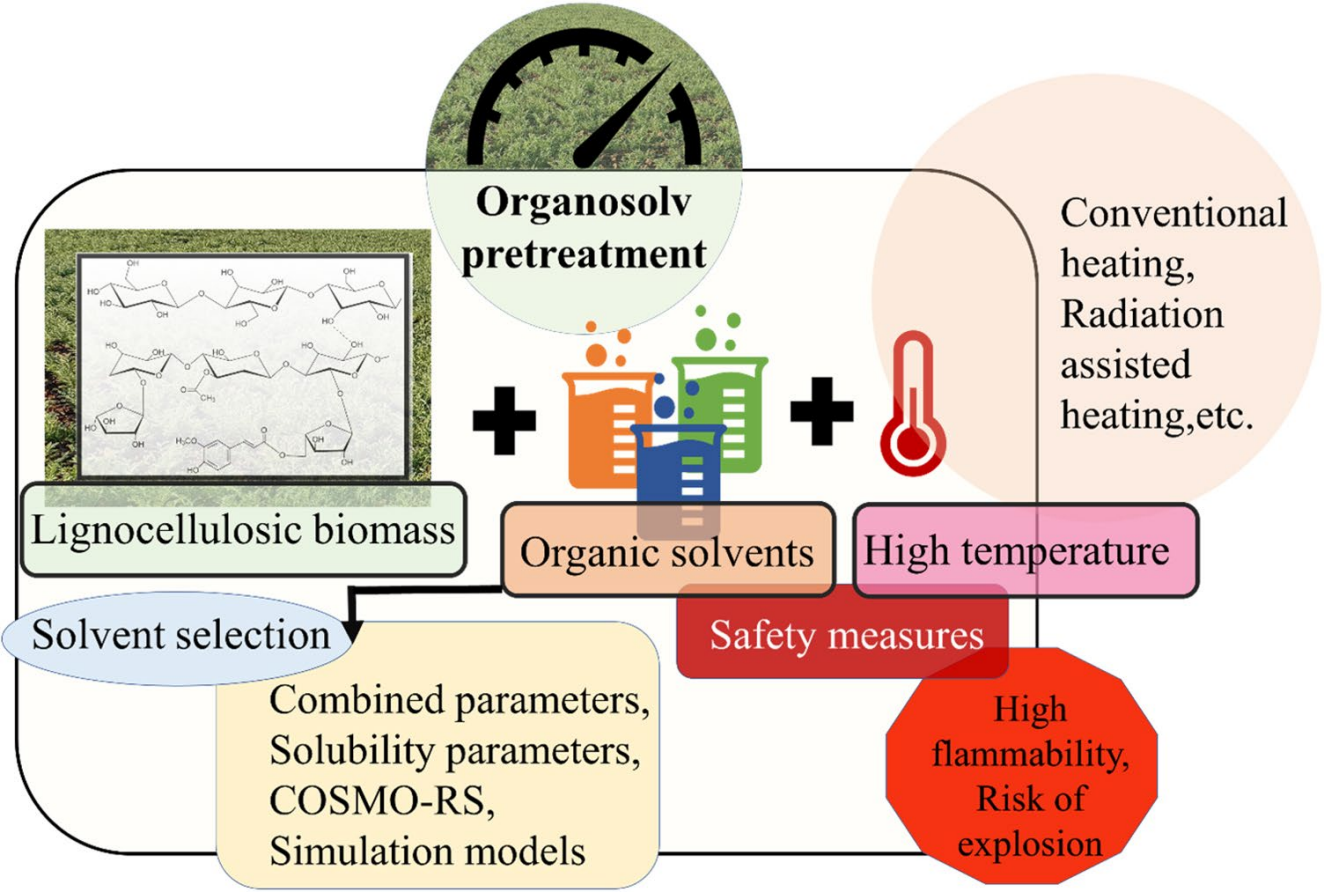

## Introduction

The lignocellulosic biomass (LCB) is an excellent substrate for its utilization in the biorefinery sector (Sheikh et al. 2013). Despite its global significance, the utility of LCB is restricted due to its recalcitrance, low efficiency of the pretreatment process, and quantity of the products (Balan 2014; Sun et al. 2022). Thus, to overcome the recalcitrance, effective pretreatment approaches have been established that allows the modification of the LCB structure (Jamaldheen et al. 2022). The pretreatment can be done using various approaches, of which, the organosolv method enables the efficient fractionation of LCB to lignin, hemicellulose, and cellulose (Ferreira and Taherzadeh 2020) can be a suitable alternative. As compared to other methods organosolv method is economically feasible as the majority of the utilized solvents in the pretreatment system could be retrieved and recycled, attributed due to their low boiling points (Borand and Karaosmanoǧlu 2018). The selection of solvents for organosolv pretreatment majorly depends on the solvent’s effect or its action to break the recalcitrance of the biomass (Shuai and Luterbacher 2016). Hildebrand and Hansen solubility parameters (HSP) have been widely utilized for the selection of a better solvent of high performance for the desired treatment of LCB. Recently, other models and methods have been used for the selection of solvents for effective delignification. They include Molecular Dynamics (MD) simulations, Quantum Chemical (QC), and conductor-like Screening Model for Real Solvents (COSMO-RS), which help in better interpretation of the solubility factors of the selected solvents (Achinivu et al. 2021). Other factors such as loss of tangent, dielectric constant, etc. have also proven to be useful for the screening of desired solvent. In addition, the efficacy of the pretreatment could be further improved by integrating organosolv with other physical systems, such as carbocation scavengers, microwave radiation, green-solvents, etc. (Chu et al. 2021; Sun 2021; Yang et al. 2020).

Over the past decade, an increase in interest was found towards biofuel sector with respect to the development of climate change mitigation policies and reduction of vehicular greenhouse gas (GHG) emission strategies. Since then, more than 60 countries have launched biofuel programs with targets for blending biofuels into their fuel pools (Jeswani et al. 2020). The global estimations by International Renewable Energy Agency-2020 (IRENA-2020) reported a total bioenergy production of ~ 115.7 GW. Furthermore, European Union (EU) accessed the highest bioenergy production at ~ 38.5 GW, trailed by Asia at ~ 36.27 GW, of which China at ~ 16.54 GW shared the maximum production of bioenergy trailed by India at ~ 10.23 GW (Ambaye et al. 2021; Hu et al. 2021). In this context, low-cost and efficient organosolv pretreatment-driven biorefineries can be beneficial for the enhancement of bioenergy production globally.

Thus, the current review provides a broad understanding of the organosolv pretreatment systems, such as organic solvents, working mechanisms, current technological updates, trends, etc. Moving on to the main objective, the review discusses different parameters of the solvents to be evaluated before tailoring specific pretreatment systems in biorefineries. The key impediments to its effective working and its challenges have also been discussed with a focus on its improvement strategies. Finally, the Life cycle assessment (LCA) and techno-economic assessment (TEA) of organosolv pretreatment systems have been compared and analysed to decode their economic and environmental contributions, for its commercialisation in biorefineries.

## Biomass and its recalcitrance: a major bottleneck

**Fig. 1:**
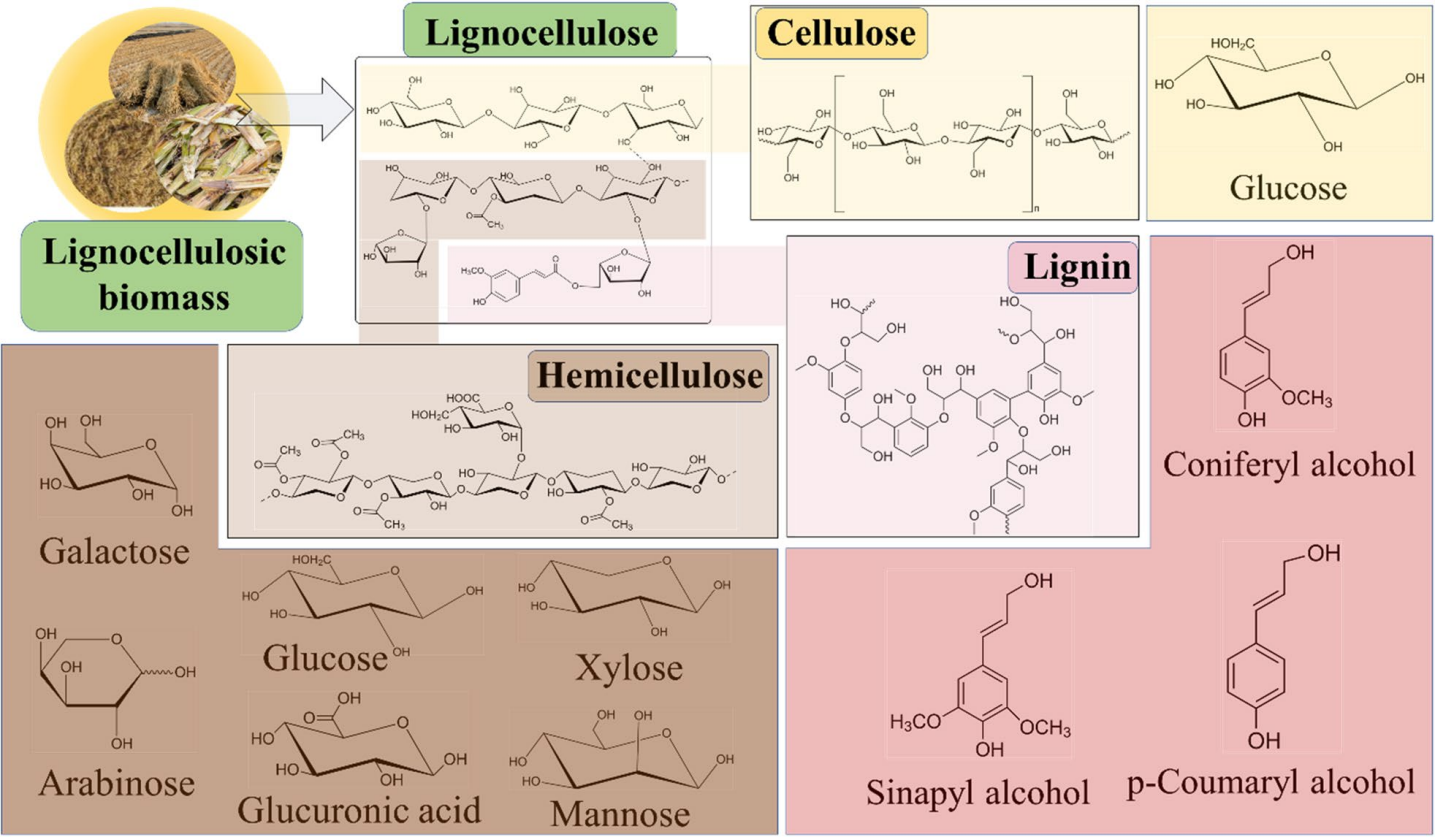
Diagrammatical representation of the chemical structures of different components in lignocellulose

The utilization of LCB for the generations of diverse biofuels is a promising concept in the frame of producing sustainable, and green energy (Potrč et al. 2021). The cellulose, hemicellulose, and lignin are arranged in complex and non-uniform three-dimensional (3D) structures (Singhvi and Gokhale 2019). The lignin component is a crosslinked aromatic polymer, where cellulose and hemicellulose form cross-links that leads to rigid structure formation. The helical structure of lignin is created by the polymerization of three monomer units of phenylpropanoid, cellulose is a semi-crystalline natural biopolymer that has both crystalline and amorphous sites and hemicellulose being a heterogeneous polysaccharide (Fig. 1).

The structural and chemical complexity of LCB results in its recalcitrance (Paës et al. 2019) of which the prior includes degree of polymerization, cellulose crystallinity, etc. and the latter includes composition, content of hemicellulose, lignin, and acetyl groups (Zoghlami and Paës 2019). The existence of hemicellulose and lignin in LCB has a great influence on its recalcitrance as they hinder the contact between cellulose and the enzyme greatly. This narrows down the enzymatic hydrolysis rate for raw LCB. The properties of lignin including the non-productive binding and enzyme inactivation also lead to lower enzymatic hydrolysis efficiency (Zhou et al. 2021).

Thus, the step of pretreatment becomes compulsory to break the open associations of polymers, which enables to access enzymes and catalysts in the LCB and aids in the conversion of polysaccharides into oligosaccharides, monosaccharides, etc. (Paës et al. 2019) (Table 1). Furthermore, delignification is also important to ease the bioprocessing of LCB. The delignification efficiency, quality as well as the amount of the valuable products, and formations of inhibitors are all dependent on the type of pretreatment employed (Jamaldheen et al. 2022)*.*

**Table 1:** Various pretreatment methods used in biorefineries and their results

Pretreatment type	Pretreatment method	Biomass used	Untreated sample	Treated sample	Remarks	References
Physical	Ball milling	Oil palm biomass	15.9% glucose yield	67.5% glucose yield	CI reduced from 56.1% (untreated) to 9.3%	(Zakaria et al. 2014)
			5.4% Xylose yield	80.1% xylose yield		
	Twin-screw extrusion	Corn stover	25 g/L glucose yield	45 g/L glucose yield	Increase in cellulose content, decrease in hemicellulose content, and CI	(Wang et al. 2020)
			19 g/L Xylose yield	40 g/L Xylose yield		
	Microwave pretreatment	Wheat straw	Ethanol yield of 26.78 g/kg	Ethanol yield of 148.93 g/kg	–	(Xu et al. 2011)
			–	Cellulose recovery > 93%		
				80% Hemicellulose recovery		
Chemical pretreatment	Alkaline pretreatment	Corn stover	–	glucose and xylose yield 0.48 g/g of the original biomass	78.2% sugar yield	(Li et al. 2012b)
				92% lignin removal		
		Corn stover	–	95.1% glucose yield	Glucose yields were two to fourfold compared to untreated, with less than 5% cellulose removal	(Mirmohamadsadeghi et al. 2016)
				Fivefold xylose yield		
				40–45% lignin removal		
		Miscanthus	–	62.3% glucose yield		
				20-fold xylose yield		
				40–45% lignin removal		
		Switchgrass	–	81.3% glucose yield		
				40–45% lignin removal		
	Acid pretreatment	Sunflower stalks	100 g raw material	33 g glucose recovery	Recovery of 65% of the glucose and xylose present in the raw material	(Ruiz et al. 2013)
				33 g xylose recovery		
	Organosolv pretreatment	Sugarcane bagasse	–	91% glucose yields	99% glucan enzyme digestibility, 90% lignin purity	(Hassanpour et al. 2020)
				67% xylose yields		
				63% lignin yield		
Physio-chemical pretreatment	AFEX	Corn stover	~ 32% glucan conversion	Higher severity AFEX gave rise to higher glucan conversion (~ 85%)	The total pore volume of the biomass decreased	(Chaudhari et al. 2022)
			~ 23% Xylan conversion	~ 85% maximum xylan conversion		
	Hot-water pretreatment	Napier grass	–	73% of inhibitor-free glucose yield	Higher temperatures may lead to inhibitor production	(Wells et al. 2020)
		Energycane	–	65% of inhibitor-free glucose yield		
	Steam explosion	Spruce wood chips	Very recalcitrant	Up to 90% cellulose digestibility	The particle size of the biomass is decreased	(Pielhop et al. 2016)
Biological pretreatment	White rot fungi (*Pleurotus ostreatus*)	*Eucalyptus grandis* sawdust	2.8% hydrolysable cellulose recovery	16.7% of total cellulose generation for hydrolysis	–	(Castoldi et al. 2014)
	White rot fungi (*Ceriporiopsis subvermispora*)	Sugarcane bagasse	–	47% of the potential glucose of untreated biomass was recovered after pretreatment followed by enzymatic hydrolysis	Increased cellulose digestibility of the biomass	(Machado and Ferraz 2017)
	Mixed microbes/bacteria	Corn straw	–	Cellulose, hemicellulose, and lignin degradation rates were 34.9%, 44.4%, and 39.2%, respectively	Strong ability to degrade lignin, could accelerate the hemicellulose degradation rate, increased methane content, and shortened the fermentation period	(Li et al. 2020)

*CI* Crystalline index

Though many pretreatment strategies have been discovered to be efficient for breaking the recalcitrance of LCB, the retrieval of high-purity lignin has only been possible mainly through the organosolv method. The method has a huge potential to release maximum sugars from LCB (Joy and Krishnan 2022). Organosolv pretreatment is known for the chemical separation of cellulose, hemicellulose, and lignin. It produces cellulose of high purity having minimum degradation rates and fractionation of hemicellulose with high efficiency (Rezania et al. 2020). Ethanol-pretreated hybrid poplar can potentially produce petroleum-derived aromatics (Bär et al. 2018). Optically pure omega-3 fatty acids with high DHA and D-lactic acid can be produced from the cellulose-rich pulp of isobutanol-pretreated beechwood (Karnaouri et al. 2021).

## Organic solvents: foundation of organosolv technology

**Fig. 2:**
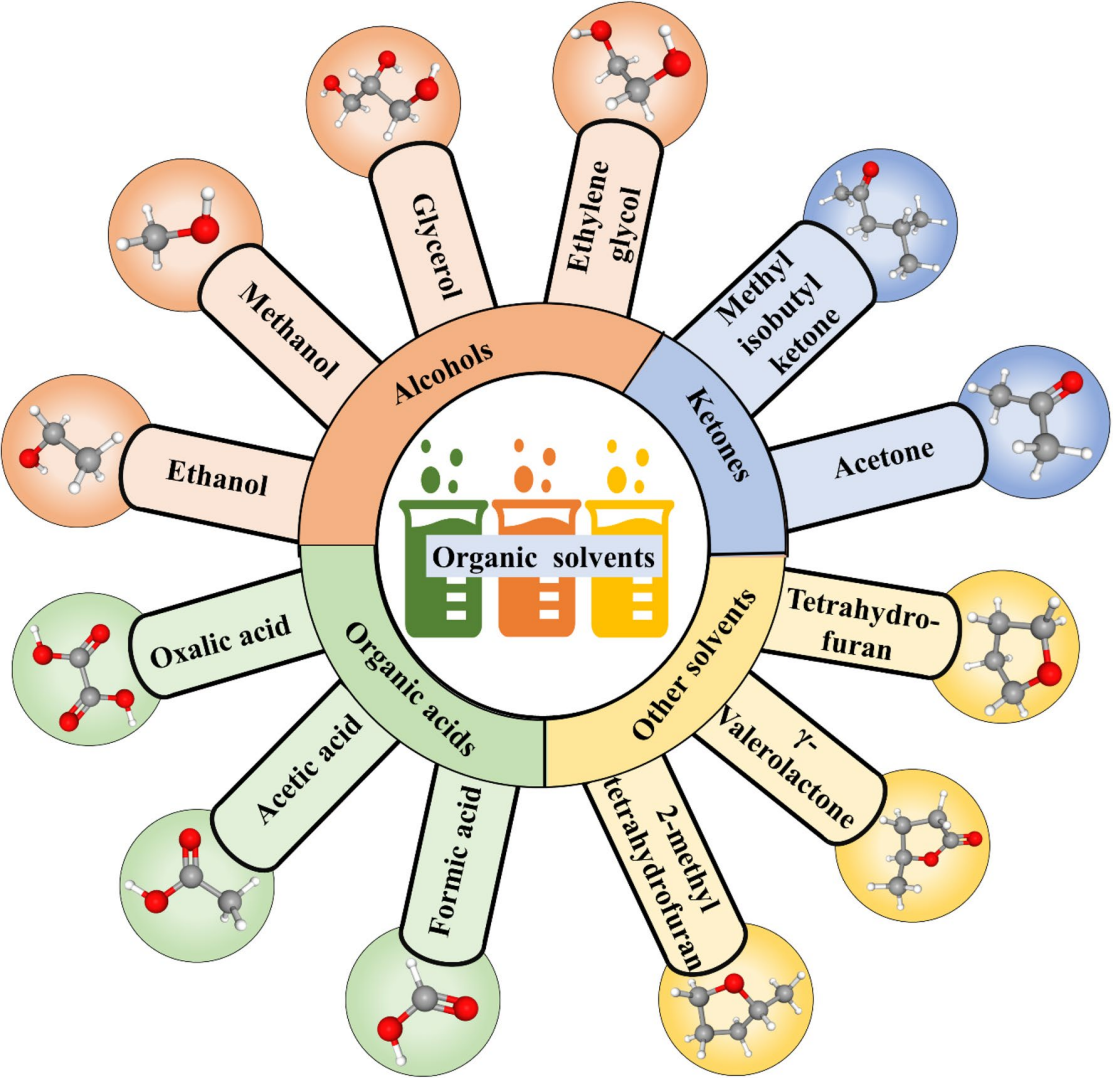
Different types of organic solvents used in the organosolv process and their 3-D conformers. (The 3-D conformers have been freely accessed from https://pubchem.ncbi.nlm.nih.gov/)

The main purpose of organosolv technology is second-generation biofuel production by enhancing lignin removal and cellulose digestibility. Over the past decade, a huge array of biomass has been treated for lignin removal by different methods. The various treatment approaches include aqueous dilute acids and organosolv treatments using alcohols, acetone, γ-valerolactone, etc. (Fig. 2) These pretreatment techniques, in the presence of water, have resulted in significant lignin removal, i.e., > 70%. In the case of dilute acids, the lignin is seldom removed/dissolved (Li et al. 2010). The treatment cleaves ether linkages and the depolymerized fragments that are water-soluble deposits on the pretreated fibres thereby hindering the further accessibility for the co-generation of desirable end products (Shuai et al. 2010; Moxley et al. 2012). Thus, steady dissolution allows condensation and depolymerization of lignin and associated protein in the presence of organosolvents, which allows the action of catalyst on the native surface of lignin thereby facilitating its removal for further action eventually allowing efficient pretreatment (Luterbacher et al. 2015a). Lignin extracted through the organosolv method exhibits high purity, narrow molecular weight, homogeneity, etc. making it suitable for industrial applications (Ramírez-wong et al. 2014). It fundamentally works by the lignin dissolution by smiting the α- and β-aryl ether linkages (Chin et al. 2020a, b) resulting in the generation of fragments of lignin, i.e., low molecular mass, and phenolics, eventually leading to the dissolution of lignin (Zhang et al. 2016b). Furthermore, Vieira et al. (2023) have reported a detailed insight into the organosolv pretreatment of coconut waste of the Scopus database. Thus, the worldwide concern of fossil fuel limitation and the potential scope of organosolv method has allowed the scientific community globally to identify various solvents (Fig. 2) and have been elaborated as follows:

### Alcohols

Alcohol due to its boiling point, i.e., low and high (LBPA and HBPA), is regarded as an efficient agent for organosolv pretreatment (Table 2). The prior enables the pretreatment to be conducted at relatively lower temperatures with easy solvent recovery and the latter has the advantage of attaining high temperatures under atmospheric pressure (Zhao et al. 2009).

**Table 2:** Pretreatment conditions, efficiency, and results of different alcohol organosolv pretreatment systems

Alcohols	Biomass	Pretreatment condition					Result	References
		Solvent concentration	Solid–liquid ratio	Temperature (°C)	Reaction time (min)	Catalyst/additional conditions		
Ethanol	Softwood	63 wt%	–	~ 235	90	Formic acid	~ 65% delignification	(Agnihotri et al. 2015)
	Sugarcane bagasse	50 wt%	–	~ 210	90–120		~ 80% delignification	
	Hybrid poplar	60%	8:1 ratio	160–180	60	H_2_SO_4_	> 75% Glucose hydrolysis yields	(Bär et al. 2018)
	Wheat straw	60%, w/w	–	200	60	–	Organosolv lignin	(De Wild et al. 2012)
	Silver birch	50% or 60%	1:10	180 or 200	15 or 30	H_2_SO_4_	Cellulose content increased from 37.1 to 69.2%, with 12.96% hemicellulose, and 13.24% lignin content	(Monção et al. 2021)
Isobutanol	Beechwood	50% v/v aqueous isobutanol	1:10	160	120	Mild acid-free oxidative conditions	High delignification (97%), oligosaccharide recovery in 43.3% aqueous fraction, 92.6 wt% cellulose-rich pulp	(Karnaouri et al. 2021)
1-Butanol	Sorghum bagasse	25%	6:80	200	60	H_2_SO_4_	84.9% and 15.3% highest cellulose and low lignin content, respectively	(Teramura et al. 2018)
Methanol	Cauliflower stalk	100%	–	121	30	Sodium acetate	77.85 ± 0.58% cellulose yield and 1.07 ± 0.71% hemicellulose yield	(Majumdar et al. 2019)
	Cauliflower leaf	100%	–	121	30	Sodium acetate	74.97 ± 0.39% cellulose yield, 6.84 ± 0.81% hemicellulose yield	
	Hazelnut skin	50% v/v	–	130, 160, 200	60	H_2_SO_4_	Lignin content reduction from 39.66 to 34.73%, increase in bioavailable sugars	(Oliva et al. 2021)
Glycerol	Eucalyptus wood	56% aqueous glycerol	1:10	200	69	–	65% lignin solubilisation, 99% cellulose retention in biomass	(Romaní et al. 2016)
	Wheat straw	70% aqueous glycerol	1:20	220	180	–	95% cellulose retention, 65% lignin removal, 70% hemicellulose removal	(Sun et al. 2015)
	Sugarcane bagasse	70% industrial glycerol	1:20	200–240	60–300	–	> 65% lignin and > 80% hemicellulose removal, > 95% cellulose retention	(Sun et al. 2016)
THFA	Hybrid *Pennisetum*	–	1:12	100	120	H_2_SO_4_	87.5% enzyme digestibility, recovered 80.8% total glucan in the untreated HP	(Tan et al. 2019)
Ethylene glycol	Degraded empty fruit bunch	50% v/v ethylene glycol	1:10	80	45	NaOH	75.1% High delignification, 81.5% hemicellulose removal, and 90.4% cellulose recovery	(Chin et al. 2020a)
	Bagasse	–	1:10	110–150	60	HCl	~ 99.3% hemicellulose and ~ 67.1% lignin removal, 94.3%, glucose recovery yield	(Wei et al. 2021)
		–	1:10	110–150	60	NaOH	~ 90.9% lignin removal and ~ 28.8% hemicellulose degradation, 92.5% glucose recovery yield	
	Rice straw	90% ethylene glycol	1:20	150	30	AlCl_3_	88% delignification, 90% hemicellulose removal, 100% cellulose recovery	(Tang et al. 2019)

In the case of LBPAs, the main solvents used are ethanol and methanol, though propanol and butanol can also be used but are usually avoided due to high solvent costs (Chin et al. 2020b). Ethanol is an excellent solvent that is beneficial in terms of low cost, less toxic nature, high miscibility with water, easy recovery, etc. (Zhang et al. 2016a). Ferreira and Taherzadeh (2020) stated that the usage of ethanol has been reinforced by paradigm shift tactics with the incorporation of first/second-generation bioethanol for promoting the commercialization of cellulosic ethanol. Gómez-Cruz et al. (2022) used exhausted olive pomace and used a two-stage extraction process to determine the influence of ethanol-oriented organosolv-assisted pretreatment on its delignification and hydrolysis. Among the various systems, ethanol (50%) catalyzed with sulphuric acid (1%) at 140 °C for 60 min with biomass (15%) enabled efficient enzymatic digestibility (81%) along with the attainment of high grade of organosolv (dissolved) lignin (71%) that were superior of guaiacyl units.

Methanol pretreatment is mainly used for the pretreatment of woody biomass (WB). It enhances the penetration of pulping liquor in LCB and increases delignification rates. Even though it is effective as a low boiling solvent in terms of the pretreatment methods, however, its toxic nature is a major bottleneck. As a noxious chemical, methanol also has high inflammable vapours even at low temperatures. Therefore, the process handling should be monitored carefully (Zhao et al. 2009). Methanol organosolv pretreatment is mostly carried out with an optimum range of temperature, i.e., 170–270 °C, with/without the addition of a catalyst. In the case of non-catalysed pretreatment, the cooking temperatures are usually raised for the attainment of self-acidification from bond cleavage. This tactic has been used successfully for the production of high-quality cellulose fibre in pulping sectors (Zhang et al. 2016a). Fan et al. (2023) proposed an experimental-based method for the effective pretreatment of LCB and its utility in the extraction of biofuel. It was stated that the non-condensed lignin production using LCB fractionation gives less yield and thus optimizing the parameters enhances the results. It also has an adverse impact on the structural condensation thus restricting its applications on value-added products formations, particularly in depolymerization. Thus, to overcome the limitation, Fan et al. (2023) used *p*-toluenesulfonic acid in methanol in the presence of microwave irradiation (MI) to get enhanced non-condensed lignin. The MI pretreatment reduced the lignin–carbohydrate complex (63.8 kJ/mol). The treatment allowed to hold 94.3% cellulose (solid part) and can be efficiently used for the production of glucose (98.5%) via enzymatic machinery.

In the case of pretreatment using HBPAs, polyhydroxy alcohols such as ethylene glycol, glycerol, tetrahydrofurfuryl alcohol, etc. were used. These alcohols, which can also be synthesised from renewable sources are great eco-friendly alternatives to synthetic chemicals used for pretreatment. A major bottleneck of using HBPAs for pretreatment is their high viscosity (Chin et al. 2020a, b). Ethylene glycol is a simple diol that is abundant in resources and has potentially enormous applications in energy, environment, etc. (Yue et al. 2012). Acid and alkali-catalysed ethylene glycol systems are both beneficial in terms of lignin removal, but the acid system was found to remove hemicellulose and lignin at ~ 99.3% and ~ 67.1%, respectively. The glucose recovery of the acid–ethylene glycol system was better than the alkali system from bagasse (Wei et al. 2021). Ethylene glycol has also been utilized for waste newspaper pretreatment and the generations of bioethanol. The results showed an increased hemicellulose (60%) and lignin (75%) removal with high enzymatic digestibility (94%). The recycling efficiency of the process was also found to be high and could be re-used four times without any significant changes in the efficiency of the pretreatment (Lee et al. 2010).

Glycerol a non-toxic organic solvent allows high-temperature processing of the pretreatment reaction to be conducted at atmospheric conditions. Usually, it is carried out using strong inorganic acid catalysts (Trinh et al. 2016). Glycerol, when used in pretreatment selectively deconstructs LCB and modifies the structure of the dissolved components by the process of glycerolysis. This mechanism helps protect the components from excessive degradation and produces lesser inhibitor components (Sun et al. 2022). Glycerol is a foremost non-toxic by-product of oleochemical industries during the transesterification of fatty acid to produce biodiesel and is available at low costs for its use in the pretreatment of LCB (Joy and Krishnan 2022), which also keeps the pretreatment costs in check.

### Ketones

The high delignification and cellulose recovery rates of ketones have led to their frequent use during lignocellulose fractionation. Acetone is used for organosolv pretreatment followed by methyl isobutyl ketone (MIBK) (Chin et al. 2020b). They have high solubility for lignin as well as lignin-based compounds (Zhou et al. 2021). Mild acetone pretreatment has shown excellent results in laboratory-scale lignin solubilisation. The replacement of ethanol pretreatment with acetone has been shown to prevent undesired ethylation of sugars and lignin. It was reported to enhance sustainability as it reduced energy demands (Smit et al. 2022). The low polarity of acetone accounts for its higher efficiency as compared to ethanol. The low boiling point of the solvent also permits easy separation and retrieval of the solvent by evaporation. It is usually accomplished at a high range of temperature, i.e., 160–230 °C, acetone-based pretreatment is considered to be an energy-demanding process. In addition, the addition of alkali as a catalyst could enhance the efficiency of the pretreatment method (Raita et al. 2017). MIBK has also been reported for the pretreatment of LCB. MIBK, a non-polar solvent forms a dual phasic layer with the polar solvent (H_2_O) throughout pretreatment. The recovered lignin dissolves in MIBK; however, the carbohydrate part dissolves into H_2_O which allows easy separation (Teng et al. 2016). The solvent properties including medium polarity, average boiling point, stability towards acid, miscibility with H_2_O, lower toxicity, high extraction ability for polar products, etc. make MIBK an excellent candidate for lignin extraction (Teng et al. 2016). The pretreatment conditions and results of ketone-based organosolv-assisted pretreatment on different LCBs are given in Table 3.

**Table 3:** Pretreatment conditions, efficiency, and results of different ketone organosolv pretreatment systems

Ketones	Biomass	Pretreatment conditions				Catalyst	Pretreatment result	References
		Solvent concentration	Solid–liquid ratio	Temperature (°C)	Reaction time (min)			
Acetone	Barley straw	50% w/w	1:20	140	20 min	–	66.7% high total xylose recovery, 75.4% enzymatic digestibility	(Salapa et al. 2018)
	*Pinus radiata* D. Don	50% w/w	1:7	183–197	4–46 min	–	Solid recoveries of 54.6% (70.9% glucan), 40% (46.3% glucan) and 36 g/L (99.5%) ethanol productivity	(Araque et al. 2008)
	Mustard	80%	1:10	121	90 min	H_2_SO_4_	32.9% lignin reduction and 13.67% maximum glucose yield	(Singh et al. 2021)
	Wheat straw	50% w/w aqueous acetone	1:10	140	120 min	H_2_SO_4_	96.8 wt% C5 sugar recovery and 91.4 wt% C6 sugar recovery 79.1 wt% delignification	(Smit & Huijgen 2017)
	Hardwood (birch)	50% w/w	1:5	140	120 min	H_2_SO_4_	87.7 wt% C6 recovery, 92.0 wt% C5 recovery, 86.4 wt% delignification	
	Softwood (pine)	50% w/w	1:5	140	120 min	H_2_SO_4_	74.2 wt% C6 recovery, 88.6 wt% C5 recovery, 31.6 wt% delignification	
Methyl isobutyl ketone–acetone–water	Corn stover	–	–	140	56 min	H_2_SO_4_	Cellulose-enriched fraction contained 88% Glucan content, lignin enriched fraction contained 85% lignin content	(Katahira et al. 2014)
	Sugarcane bagasse	–	1:40	180	15–60 min	Acidic ionic liquid catalyst	Highest conversion of bagasse (85.8–92.7%) and the 76.3% lignin extraction ratio	(Teng et al. 2016)

C5 sugar–arabinose and xylose; C6 sugar galactose, glucose, mannose, and rhamnose

### Organic acids

Acetic acid pretreatment is performed generally under high temperatures of 135–200 °C, with short retention times of 30–120 °C. Reportedly, the range of temperature in the process can be minimised to 110 °C, by adding sulfuric acid (H_2_SO_4_) (Chin et al. 2020b). The lignin derived from the acetic acid organosolv pretreatment is known to have many important applications as it has properties, such as low molecular weight and high reactivity (Tang et al. 2017). Other organic acids used for pretreatment purposes include oxalic acid, formic acid, etc. In general, oxalic acid has been utilized as a substitute for H_2_SO_4_ which is also used as an acid catalyst during pretreatment. It is an environmentally friendly method producing very minimal concentrations of fermentation inhibitors. It is also known to have greater catalytic efficiency than H_2_SO_4_ (Kundu and Lee 2015). Studies were conducted on poplar wood using oxalic acid pretreatment at 160 °C for 20 min. From the liquid fraction of pretreated poplar wood, ~ 40.22 g/L fermentable sugars were retrieved and utilized for subsequent saccharification and fermentation (SSF) to generate bioethanol (8.6 g/L) (Kim et al. 2009). In addition, recently Sar et al. (2022) used oak husk and provided ethanol organosolv treatment, where H_2_SO_4_ was substituted by phosphoric and oxalic acids and various parameters were explored using a one-factor-at-a-time approach. It was reported that the deduction in washing with solutions, i.e., water/solvent/both did not affect glucan recovery but did impact lignin recovery negatively. The optimum treatment and glucan recovery were attained solid:liquid ratio of 1:2 using aqueous ethanol (50% v/v) acidulated with oxalic acid at 210 °C for 90 min. This approach of treatment can perhaps be utilized for the development of essential valuable goods, eventually contributing to the future of bioeconomy (Table 4).

**Table 4:** Pretreatment conditions, efficiency, and results of different organic acid pretreatment systems

Organic acids	Biomass	Pretreatment conditions				Pretreatment result				References
		Solvent concentration	Solid–liquid ratio	Temperature (°C)	Reaction time (min)	Cellulose content	Hemicellulose content	Lignin content	Others	
Acetic acid–formic acid	Wheat straw	65/35 mass ratio, 85% in water	0.96:10	105	180	63% and 93% of cellulose purity and recovery	58% wheat straw xylan	78% and 84% lignin purity and recovery	44% proteins, 69% minerals, etc	(Snelders et al. 2014)
Formic acid	Sugarcane tops	85%	1:7.5	125	90	84.7% cellulose retention	~ 96.3% hemicellulose removal	~ 90.8% lignin removal	–	(Pathak et al. 2021)
	Bamboo	85%	1:7	145	45	42.2% cellulose pulp	8.5% hemicellulose-rich fraction	31.5% lignin-rich fraction	3.56% furfural and 3.80% acetic acid	(Zhang et al. 2018)
Oxalic acid	Corn cob	–	1:20	140	150	–	85.0% yield of xylose	–	178.93–69.76 mg/g total sugar content	(Cheng et al. 2018)
	Yellow poplar	82 mM	1:8	160	58	64.09% glucan	–	25.16% lignin composition	High ethanol yield 34.54 g/L	(Jeong and Lee 2016)
	Cassava stem	1% w/v	1:10	121	15	–	–	–	1.509 g/L Total reducing sugar	(Sivamani and Baskar 2018)
	Maple wood	0.5%		160	27.5	1.7% glucose yield	87.5% total xylose yield	–	–	(Zhang et al. 2013)
Acetic acid	Forest residues	50% v/v	1:10	190	60	31.2–40.3% cellulose	16.2% hemicellulose	–	12.2–46.7–52.5% total carbohydrates	(Kabir et al. 2015)

### Other solvents

Recently, other solvents such as 2-methyl tetrahydrofuran (2-MeTHF), tetrahydrofuran (THF), γ-Valerolactone (GVL), etc. have also been investigated for the organo-assisted LCB pretreatment (Chin et al. 2020b; Raj et al. 2021). A lower boiling point of THF (66 °C), a cyclic ether, enables its recovery after the pretreatment even via simple distillation. The ability of high-purity solvent recovery (97%) is due to THF’s high azeotropic concentrations with water (95%). THF contains CH donor, ether acceptor properties, etc. On mixing with water, it creates a solvent environment with an amphiphilic character (Chin et al. 2020b). The two dissimilar solvents species thus accumulate into different structures of lignin. Reducing the H_2_O content up to 25% v/v increased the rate of delignification, but a further reduction of water contradicted the result. The solvent could also be used under low temperatures (< 75 °C) (Thoresen et al. 2020).

2-MeTHF is a solvent that has little miscibility with H_2_O, but its constancy is greater than reported solvents, such as THF. This paves the way for utilizing the 2-MeTHF as a green organic solvent as well as a potential replacement for THF (Ashok et al. 2022). Thus, 2-MeTHF is an ecologically friendly alternative to polar and aprotic solvents with low polymerization tendency, high stability in acid and base solutions, etc. It is also a less volatile liquid which adds up to improved safety and recovery efficiencies (Viola et al. 2021). When used in pretreatment systems, 2-MeTHF with oxalic acid under microwave-assisted fractionation led to a 92.89% cellulose hydrolysis rate (Li et al. 2017). GVL is also a biomass-derived solvent which is an outstanding substitute for conventional organic solvents due to its less-toxic, renewable nature. It can also be reused subsequently via the extraction process (Gelosia et al. 2017). GVL is usually used for mild LCB pretreatment. In a study using 80% GVL pretreatment at 120 °C, acid loading with H_2_SO_4_ on hardwood reportedly removed 80% lignin from it. The cellulose retention was 96–99% in the pretreated substrates (Shuai et al. 2016). It also reduces the thermal stability of the pretreated residues (Tan et al. 2019).

## Organosolv pretreatment mechanism

**Fig. 3:**
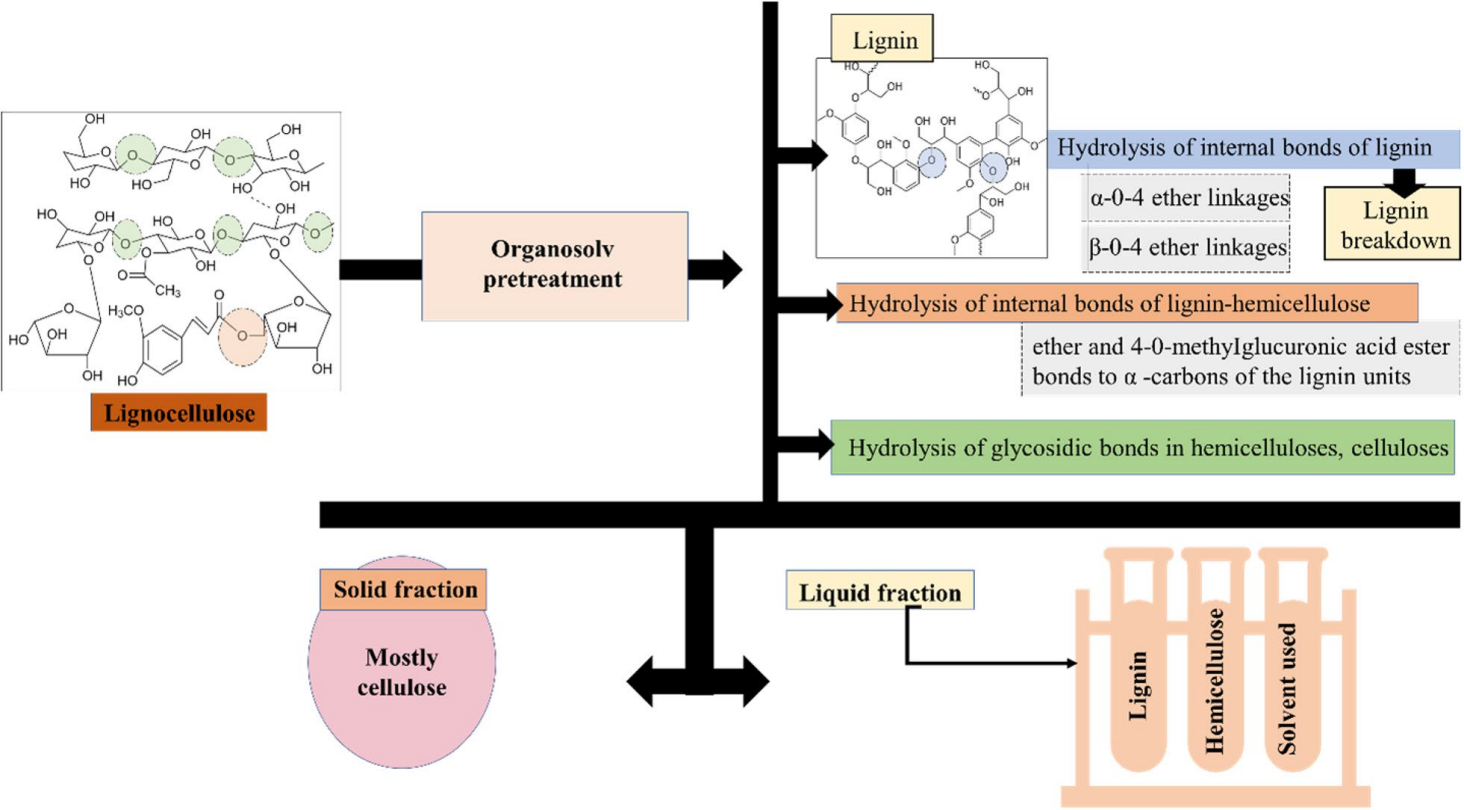
Diagrammatical representation of the mechanistic action of organosolv pretreatment on lignocellulosic biomass

Proposedly, the major mechanism of the organosolv process is the cleavage of α- and β-aryl ether linkages, which leads to the disintegration/dissolution of lignin (El Hage et al. 2010). The α-aryl ether linkages have extra susceptibility to disruption rather than stable β-aryl ether linkages. According to McDonough (1992b), the three pathways have been associated with the cleavage of α-aryl linkages, i.e., (i) solvolytic cleavage by SN2 nucleophilic substitution, and formation; (ii) quinone methide intermediate, and (iii) benzyl carbocation. Though the acid catalyst additions may not induce significant changes in the reaction mechanism, it is reported that the cleavage of β-aryl ether linkages is likely enhanced, foremost delignification. The pathways of β-aryl ether linkages’ cleavage include (i) solvolytic cleavage and elimination of formaldehyde, (ii) homolytic and solvolytic cleavage for the formation of Hebbart ketones, and (iii) benzyl carbocation formation (McDonough 1992a; Chin et al. 2020b). The action mechanism of lignocellulose breakdown in the organosolv process is diagrammatically represented in Fig. 3.

The organosolv treatment is primarily targeted for lignin removal and consists of the cleavage of ether linkages ensuing the generation of lignin fragments with a low molecular mass, phenolics, and lignin dissolution. The amount of lignin in LCB is responsible for the recalcitrance nature thus restricting the exploration of its full potential in biorefinery. The elimination of lignin allows the retrieval of underlying treasure of polysaccharides along with easy access to enzymes for the development of valuable products (Mansfield et al. 1999). After lignin removal, cellulase can easily bind to the native and residual lignin (Yang et al. 2011; Kumar et al. 2012) and the addition of non-saccharifying enzymes will improve the hydrolysis of the LCB as it will allow the cocktail of non-saccharifying enzymes to bind to lignin rather than cellulase binding (Wang et al. 2013; Harrison et al. 2014). Thus, it can be inferred that lignin removal not necessarily will enhance the saccharification but minimizing the non-specific interaction of enzymes on the lignin surface shows a noteworthy role in improving the enzymatic saccharification (Nakagame et al. 2011; Lou et al. 2013).

**Fig. 4:**
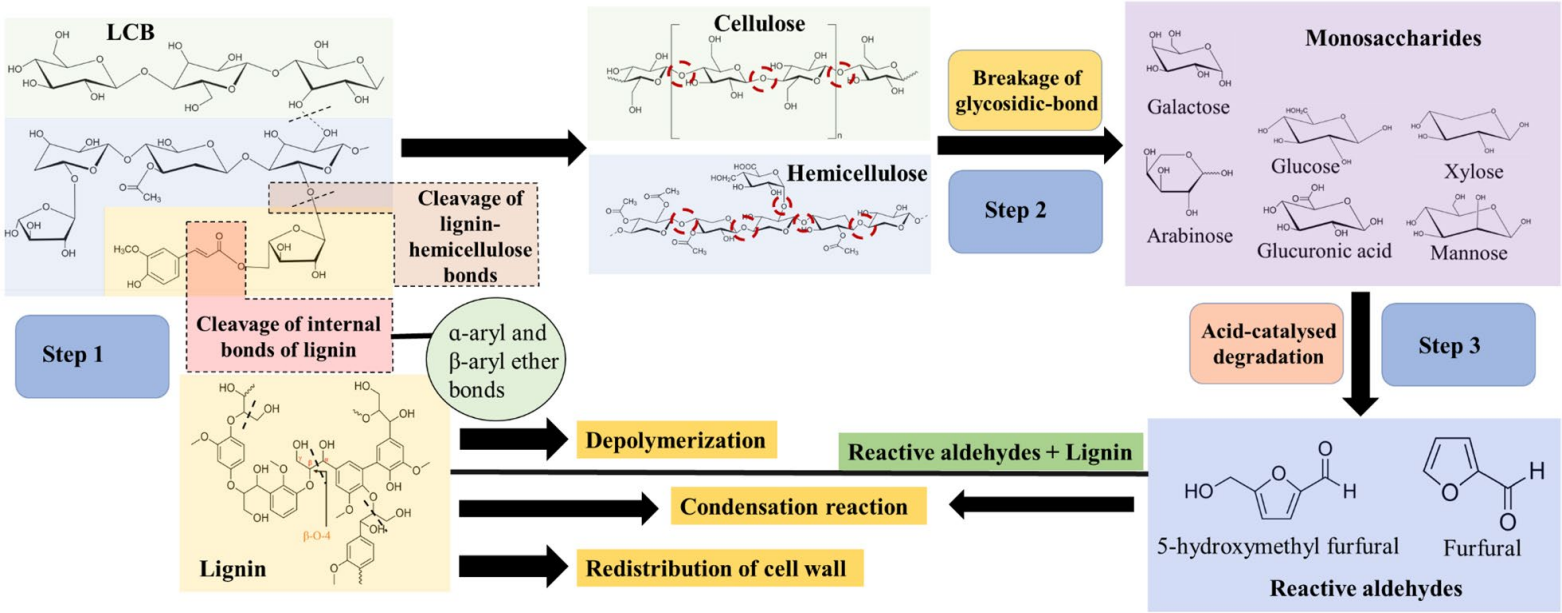
Diagram demonstrating the reaction mechanism of alcohols with lignocellulosic biomass

In the case of organosolv pretreatments involving alcohol, the mechanism of LCB breakdown proceeds through three major chemical reactions (Fig. 4). The first reaction involves the hydrolysis of the internal lignin and bonds of lignin–hemicellulose. The second reaction is glycosidic bond hydrolysis in hemicellulose and also to a little extent in other sugar compounds (cellulose) with respect to the conditions of the reaction. The third reaction step is the breakdown of monosaccharides into 5-hydroxymethyl furfural and furfural using acid catalysis. This is also followed by the condensation between lignin and reactive aldehydes (Chum et al. 1985; Zhao et al. 2009). The lignin counterpart in alcohol pretreatment frequently undergoes (i) depolymerization, (ii) condensation, and (iii) redistribution (Zhao et al. 2017).

**Fig. 5:**
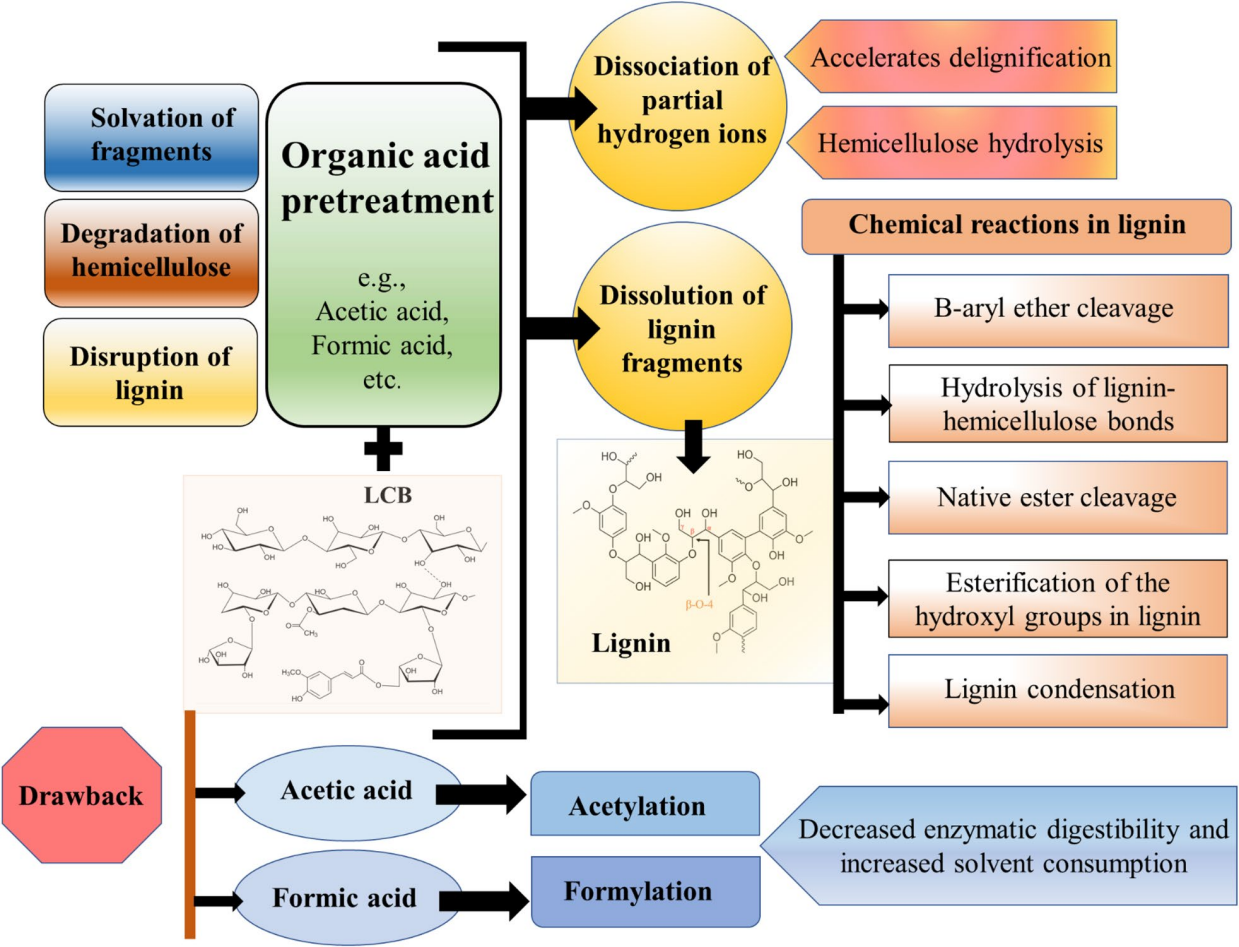
Diagrammatic representation of reaction mechanism of organic acids with lignocellulosic biomass

Similar to the alcohol-assisted pretreatment, the reaction mechanism of organic-acid pretreatment is a combination of two steps, i.e., (i) disruption of lignin, and degradation of hemicellulose and (ii) fragments solvation (Fig. 5). The organic acids enable the detachment of partial hydrogen ions to boost the delignification as well as hemicellulose hydrolysis. It also dissolves the lignin fragments (McDonough 1992b; Chin et al. 2021). Organic acids such as formic acid and acetic acid are known to have good solvency against lignin (Zhao et al. 2009). In general, the biochemical reactions of lignin breakdown involve the condensation of lignin, β-aryl ether cleavage, native ester cleavage, lignin–hemicellulose bonds hydrolysis, and hydroxyl groups esterification in lignin (Li et al. 2012a). However, in this pretreatment, formylation and acetylation of cellulose take place, which results in decreased enzymatic digestibility and increased solvent consumption (Young et al. 1986; Zhao et al. 2009).

In a conventional ionic liquid process, the LCB biomass is dissolved, whereas in respect of ionic cation liquid, it forms a strong H-bond with the OH-group and thus breaks the H-bond in cellulose (Brandt et al. 2013). The organic solvent pretreatment along with pH performed a very vital role in the rate of lignin dissolution, fragmentation of lignin, and condensation of lignin. If a catalyst has not been added for the organosolv pretreatment, then the process initiates via the autoionization of H_2_O. The acetic acid and hydronium ion which are discharged from the hemicellulose allows the cleavage of α- and β-aryl ether linkages in lignin facilitating lignin breakdown. In addition, the α-ether cleavage dominates as compared to the latter, and γ-ether linkages play an insignificant/minor role in the lignin delignification (Zhang et al. 2016b; El Hage et al. 2010). Notably, the organosolvent-assisted pretreatment in the occurrence of an acid catalyst works using a similar mechanism that happens in the absence of a catalyst, but with 100 times more efficiency (Zhang et al. 2016b) and with initial acid concentration being high, the β-aryl ether bond cleavage amplifies (Wyman 2013). In addition, to delignification, hydrolysis of hemicellulose (amorphous) and a small section of cellulose (mostly amorphous) occurs that reduces the degree of cellulose polymerization and this elimination of amorphous components further allows enhanced cellulose accessibility (Zhang et al. 2016b).

Various studies have been conducted using methods such as Nuclear Magnetic Resonance (NMR), Fourier-Transform Infrared spectroscopy (FTIR), X-Ray Diffraction (XRD), and Scanning Electron Microscopy (SEM), etc. for a deep understanding of how organosolv pretreatment impacts the LCB structure. These studies led to the formulation of mechanisms of the old as well as new organosolv methods using different solvents and catalyst combinations (Ferreira and Taherzadeh 2020). The addition of a catalyst has also been shown to have a considerable impact on the delignification process by the organosolv method. When the catalyst is not added to the system, the pretreatment starts with the self-ionization of H_2_O. As a result, ions of hydronium and acetic acid, (which is liberated from the hemicellulose) act as catalysts thereby promoting the hydrolytic cleavage of both α- and β-aryl ether linkages of lignin. Moreover, the γ-ether linkage cleavage has only very little effect on lignin dissolution. Due to the existence of an acid catalyst, the mechanism is likely to be that of the absence of a catalyst, but with enhanced reaction rates. The addition of catalysts increases the cleavage of β-aryl ether linkages (Zhang et al. 2016b). Rather than the delignification process, in the acid-catalysed organosolv process, amorphous hemicellulose hydrolysis and a smaller portion of cellulose (generally amorphous) takes place. In addition, the process reduces cellulose-polymerisation. These processes altogether enhance cellulose accessibility. Therefore, solvents with the ability to (i) dissolve the lignin, avoid its condensation, (ii) improve the swell-up of the biomass, and (iii) positively react with cellulose, need to be selected for better accessibility of cellulose after acid-catalysed organosolv pretreatment of LCB. In addition, they are known to reduce pretreatment reaction times and temperatures without sacrificing the yield of glucose (Zhang et al. 2016b). Though the add-on of acid substances is highly valuable for use in organosolv pretreatment, the generation of inhibitors, corrosion of instruments, etc. raises concerns about its usage (Zhong et al. 2018).

Alkaline catalyst addition has also shown a considerable increase in the delignification rates and sugar yields in LCB. Alkalis such as NaOH, ammonia, tri-ethylamine, etc. could be employed as catalysts (Raita et al. 2017). In alkaline catalysed systems of the organosolv process, the α-ether linkages are generally cleaved if their units have phenolic groups. Here, the ionization of free phenolic groups leads to its conversion to quinone methide, which is followed by the elimination of a leaving group, positioned adjacent to the side chain. The fragmentation of lignin can occur due to the existence of α-substituent in the aroxy group of adjacent lignin. On the contrary, the units consisting only etherified phenolic hydroxyl groups cannot convert themselves into quinone methides resulting in no cleavage. The cleavage of β-O-4 linkages results in lignin depolymerization and the development of new hydroxyl groups, that makes them hydrophilic. The β-ether cleavage does not necessarily require free phenolic hydroxyl groups on benzene rings. In units that essentially have a free OH^−^ group, primarily, the α-O-4 linkage cleavage is usually followed through the nucleophilic addition of OH^−^ ion to the quinone methide formed as a result in the α-position. It is then displaced by the neighbouring β-ether substituent (McDonough 1992a; Zhao et al. 2017). Zhong et al. (2018) exhibited that the addition of alkaline and reductive hydrazine hydrate in the ethanol-based organosolv pretreatment of corn stover exhibited a high rate of delignification, i.e., 77.94%, and an improved yield of sugar, i.e., 90.27%, respectively. The proposed reaction mechanism suggests the release of hydroxide ions by the dissociation of H_2_O and the protonation of hydrazine hydrate. The OH^−^ is supposed to be mainly accountable for triggering the saponification in the ester bonds between hemicellulose and lignin, resulting in the removal of lignin. NH_3_^+^–NH_2_ which be in its bronsted acid form acts as acidic catalyst for the hydrolysis of the ester linkages of ferulate and coumarate lignin linkages through ammonolysis. In addition, –NH_2_ groups (electronegative) and the OH^−^ groups of cellulose could develop hydrogen bonding networks and eventually, the swelling up of cellulose parts.

## Organic solvents: pretreatment effect on LCB

The use of biomass in biorefinery is the most appropriate and relevant initiative towards a sustainable future. However, as known, the recalcitrant lignin is the cause of chief hindrance to the complete consumption of cellulose. To enhance the cellulose accessibility in many cases, partial removal of lignin, hemicellulose and depolymerization of cellulose has been explored (Luterbacher et al. 2013, 2015b). Furthermore, the lignin removal/delocalization could lead to the development of micro and macro pores, as it primarily allows the enzymatic machinery to access the cellulose counterpart (Xu et al. 2012; Rahikainen et al. 2013). The γ-Valerolactone has also been reported for providing efficient lignin removal (Shuai et al. 2016). In addition, the polar and non-polar functional entities impart lignin medium polarity. Thus, lignin removal can be accomplished via two steps, i.e., depolymerization of lignin and its dissolution in medium polarity solvent. Regarding the pretreatment with aqueous dilute acid, rather than initiating lignin removal, the aqueous part facilitates the deposition and self-condensation of the depolymerized lignin on the surface thereby restricting efficient enzymatic hydrolysis (Li et al. 2010; Moxley et al. 2012). Thus, the solution would be depolymerization using the organosolv approach, and the continuous dissolution of fragments of lignin condensation allows the catalyst to move to the lignin surface thereby facilitating its efficient removal.

After the lignin solubility, the other important aspect of biomass to be explored is cellulose. Various ionic liquids have been reported to efficiently dissolve the cellulose component of LCB and the mechanism of dissolution is similar to that used in textile industries (Kilpeläinen et al. 2007; Zhao et al. 2013). Fundamentally, the strong cations and anions in the solvent disrupt the H– bond and bind with the OH^−^ of cellulose to form an even stronger H– bond over the cellulose’s original structure (Lavoie et al. 2011). The anionic form affects the dissolution of cellulose as discussed previously and the cations have an impact on cellulose solubility. It can be impacted by three factors, i.e., the nature of the side chain, functional group, and cation size (Badgujar and Bhanage 2015). The treatment with ionic liquid despite being efficient has limitations such as the presence of water as it can adversely impact the H– bond formation between ionic solvent and OH^−^ of cellulose On the contrary, it was also suggested that a minimal water requirement was required for the dissolution of LCB (Brandt et al. 2010; Swatloski et al. 2002). Thus, it can be inferred that depending on the ionic solvent the water content may vary (Brandt et al. 2011).

## Current updates of the organosolv system

**Fig. 6:**
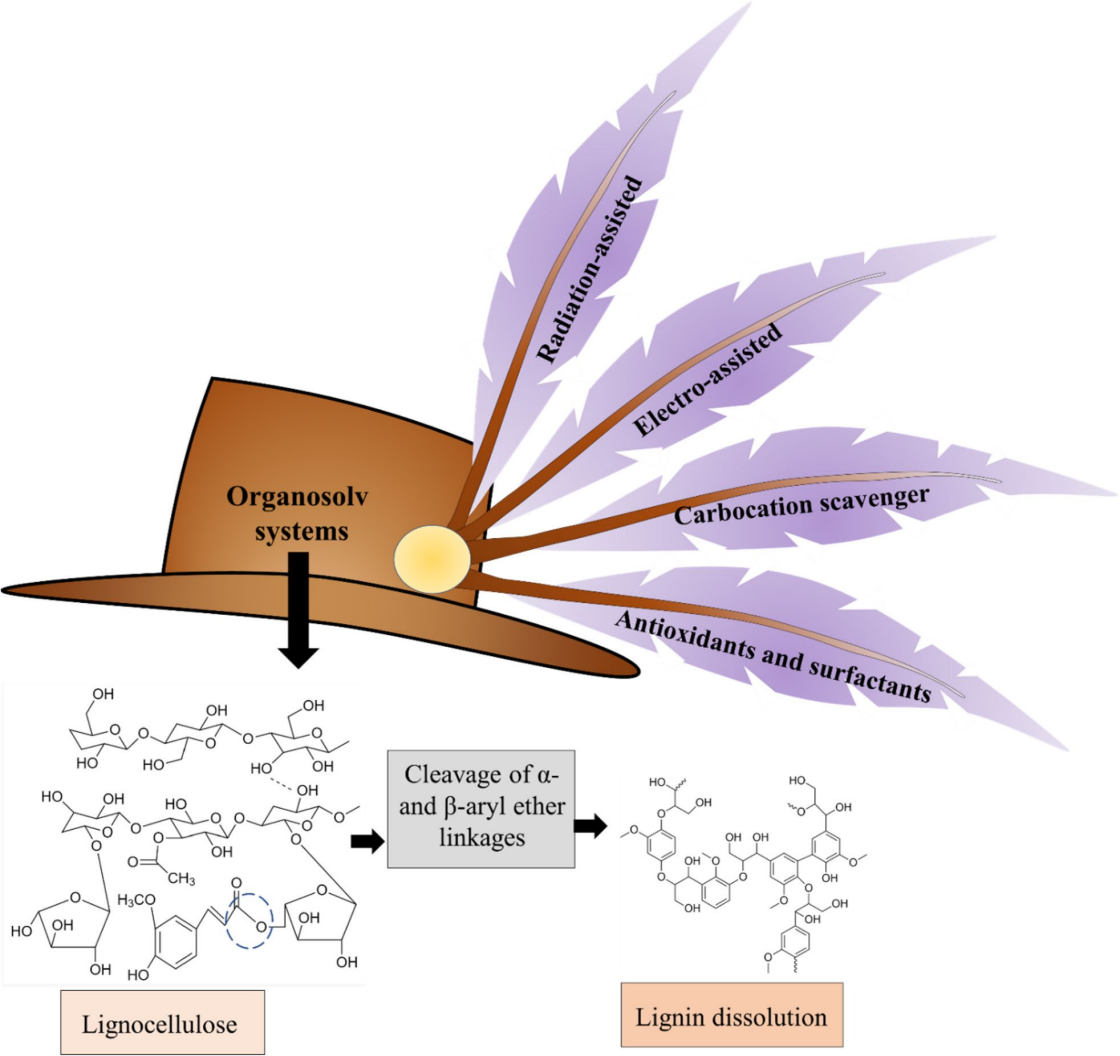
Pictorial representation of the organosolv system and the different assistance methods available currently

During organosolv pretreatment, usually, solvents of high performance for LCB are used. Recent research reports the use of innovative technologies to increase the performance of organosolv systems. Some of them include switching the conventional heating using ultrasound and microwave heating (Yang et al. 2020), electrical energy, etc. (Sun 2021) (Fig. 6).

### Radiation-assisted heating of organosolv

As the organosolv process requires heating up of the organic solvents at high temperatures, conventionally, heating methods such as oil bath is used. Such methods of heating are energy exhaustive due to the need for high temperature, pressure, and reaction times (Avelino et al. 2019). Using radiation such as the microwave was shown to considerably decrease the reaction time as well as improve the overall effectiveness of the suggested method (Alio et al. 2019). The application of microwave irradiation on the organosolv system causes the interaction of the molecular dipoles that exist in the system having the electromagnetic field of the microwave. This causes the dipoles to rotate and develop friction which results in loss of energy due to the heat release by friction through molecular rotation. The increase in effective productive collisions between the reactant molecules increases the reaction speed and also provides higher lignin yields in respect of the traditional approaches (Avelino et al. 2019). In addition, the athermal effect resulting from microwave radiation possibly breaks the hydrogen bonds between the intramolecular and intermolecular chains, which enhances the solubility as well as the conversion of the LCB in the organic solvents (Yang et al. 2020). Studies have shown that this method is effective for wood biomass fractionation with high cellulose recovery (80%), purity (70%), and lignin recovery (45%) (Alio et al. 2019). Reports have also shown that the microwave-assisted organosolv (glycerol) pretreatment of agriculture and forest leftover considerably increases levoglucosan yields. In addition, the yield of microbial inhibitors was also found to be substantially reduced by the process (Zheng et al. 2016). In another study, microwave-assisted ethanol organosolv pretreatment was applied on shells of pistachio and cherry tree pruning followed by enzymatic hydrolysis of cellulose. The system was augmented (optimized) through the CCD–RSM and the optimal conditions included 67% of ethanol–aqueous solution at 150 °C for 30 min giving 81.1% and 90.1% cellulose for shells of pistachio and cherry tree pruning, respectively. The cellulose after hydrolysis gave a glucose yield of 70 and 100 kg/m^3^ for cellulose for shells of pistachio and cherry tree pruning (Corsi et al. 2022).

Ultrasound has also been used as an auxiliary energy source for the intensification of pretreatment (Ong and Wu 2020). The incorporation of ultrasound radiation with the organosolv method was found to significantly impact and disrupt the LCB structure by the breakdown of lignin and hemicellulose bonds (Lee et al. 2020). It is suggested that ultrasound works under the principle of cavitation and acoustic streaming when incorporated into liquid slurries. The cavitation generates bubbles resulting in a rise in both temperature and pressure in the area of cavitation. In addition, the development of strong hydromechanical shear forces disrupts the cells of the materials in the cavitation area (Ofori-Boateng and Lee 2014). An ultrasound-assisted organosolv method on palm oil empty fruit bunches showed an improved reducing sugar recovery without the use of a catalyst as compared to conventional organosolv treatment with a catalyst (Lee and Ng 2019).

### Electro-assisted organosolv pretreatment

The electro-assisted organosolv pretreatment (EAOP) is a modified process that is performed under the presence of electrical energy and is viable at room temperatures. This method integrates the organosolv pretreatment with the electrochemical conversion (Sun et al. 2021; Sun and Othman 2019). Usually, ionic liquid-promoted organic solvents are used for EAOP due to their property of electrical conductivity. They are prepared by mixing ionic liquids with selected organic solvents (Sun et al. 2020). A researcher reported a modified EAOP system, wherein a binary solution of GVL (organic solvent) and 1-butyl-3-methylimidazolium acetate (ionic liquid) was used. Electrical energy was provided to the solvent through the introduction of carbon electrodes to the system. The results showed that selective fractionation of lignocellulosic materials was achievable even at low temperatures (Sun and Othman 2019). Wu et al. (2022) used integrated sweater hydrothermal with succeeding alkaline hydrogen peroxide pretreatment on corn stover. In the prior step at 190 °C and 40 min, the complex ions such as Mg^2+^, Ca^2+^, and Cl^−^ depolymerized xylan to xylo-oligosaccharides and the latter allowed successful delignification. This strategy resulted in a glucose yield of 91.16%, which makes it an excellent pretreatment method for corn stover. In addition, Yu et al. (2022a) used electrochemical pretreatment used for producing bio-oil from corn stalks. The seawater acted as an electrolyte and various parameters were investigated which led to the conclusion that lignin was efficiently deconstructed; thereby exposing cellulose and hemicellulose at 3.5% sodium chloride, 15 V for 5 h as residual solids. In addition, levoglucosan and furfural boosted the pyrolysis of bio-oil reaching 23.22% and 14.14%. Thus, the use of green pretreatment methods allows efficient upgradation of the system and enhanced end-product production.

### Carbocation scavenger—organosolv system

After pretreatment, a certain amount of lignin is known to remain inside the pretreated biomass which hinders through enzyme-assisted hydrolysis due to physical blocking and non-productive enzyme binding. A study using a carbocation (2-naphthol) and organic solvent (ethanol) complex was used to study the mitigation of such surface lignin and was discovered that the system successfully suppressed lignin condensation rates. Moreover, 2-naphthol-modified lignin showed lesser inhibition to enzyme-assisted hydrolysis (Wang et al. 2022). It was suggested that during ethanol organosolv pretreatment, lignin repolymerization was caused due to carbocation-assisted condensation and radical-assisted coupling of lignin fragments. Additives such as syringic acid and 2-naphthol are also known to forage this carbocation or radical intermediates, thus decreasing lignin polymerisation and leading to an increased hydrolysis rate. It was found that 2-naphthol added to an acid organosolv pretreatment under this principle culminated in elevated levels in lignin removal rate, i.e., 76%, and cellulose hydrolysis, i.e., 85%, respectively (Chu et al. 2021). Similarly, the addition of 3-hydroxy-2-naphthoic acid (3H2NA) with the organosolv system was beneficial for the pretreatment of various residues of agricultural waste. In corn stover, the system reportedly enhanced the 72 h hydrolysis yield by 24.08% (Liu et al. 2022).

### Addition of antioxidants and surfactants

The effectivity of using antioxidants and surfactants for effective lignin removal has also been reported. Some examples include 3-tert-butyl-4-hydroxyanisole (BHA), tween 80, tween 20, Tert-butylhydroquinone (TBHQ), and methyl-3,4,5-trihydroxybenzoate (methyl gallate), etc. Reportedly, the effects of antioxidants and surfactants in combination with organic solvents used for pretreatment showed no interference with hemicellulose removal but influenced cellulose removal. Though the antioxidants had influenced lignin removal rates, there was no considerable change in the case of surfactant addition (Schmatz and Brienzo 2021). The use of antioxidant butylated hydroxytoluene (BHT) with ethanol pretreatment was found to result in improved pretreatment resulting in 72.45% and 45.28% hemicellulose and lignin removal compared to ethanol pretreatment without BHT addition (35.09% and 56.31%, respectively) (Schmatz and Brienzo 2021). A series of experiments were conducted by Schmatz et al., (2022) using different anti-oxidant additives (TBHQ, methyl gallate, BHA, dimethyl sulfoxide, tween 20, and tween 80) in organosolv pretreatment conducted on sugarcane bagasse. It was reported that the lignin removal rates were considerably higher as compared to the organosolv process without any additives. Moreover, the addition reaction using tween 80 led to the recovery of a material that was nearly completely cellulose and resulted in high conversion rates during enzymatic hydrolysis. BHA added organosolv process resulted in a 71% lignin removal (Schmatz et al. 2022).

## Solvent selection parameters in the organosolv methods

The selection of an efficient solvent is a very important step before proceeding to the organosolv method. For the pretreatment system to provide good results, it is to understand that the solvent must have high-efficiency conversion rates to reduce the intensive energy and solvent expenditure. Some of the parameters used in the selection of the solvents in the organosolv method include combined severity factors, solubility parameters, COSMO-RS, simulation models, loss tangent, etc. (Table 5).

**Table 5:** Different selection parameters for organic solvents used in organosolv pretreatment and their significance

Parameters		Constituents/definitions	Method of calculation	Significance
Combined severity factors (CSF)		Combination of reaction time, temperature, the addition of catalysts, pH, etc	$\mathrm{Log}\left(R0\right)=\frac{\mathrm{Log }(t\mathrm{Exp}(T-T\mathrm{ref}))}{14.7}$ $\mathrm{CSF}=\mathrm{Log} \left(R0\right)-\mathrm{pH}$	• Log(*R*_0_) range of 0.5 to 0.7 was most suitable for pretreatment • A CSF value between1.0 to 2.5 is optimised for good cellulose recovery as well as improved enzymatic digestibility
Solubility parameters	Hildebrand Solubility parameters (*δ*)	The solubility of a polymer is described as the square root of the cohesive energy density	$\Delta =\sqrt{\frac{E}{\mathrm{Vm}}}$	• Gives an idea about the action of solvents in the dissolution of lignin
	Hansen Solubility parameters (*δ*)	Measures solubility of polymers using the square root of the sum of the quadratics of the three interactions (*δ*_d_, *δ*_p_, *δ*_h_)	Relative energy difference $\mathrm{RED}= \frac{\mathrm{Ra}}{R0}$	• RED ≤ 1 shows high solvent–solute affinity and can be used for pretreatment • RED˃1 shows a low affinity of the solvent with the solute
Conductor-like Screening Model for Real Solvents (COSMO-RS)	–	Combination of COSMOS with statistical thermodynamics	The *σ*-profile of the molecule is outlined from the histogram of the charge intensities around each molecule from the 3-D distribution of polarization charges	• Explains the affinity biomass–solvent interaction in a mixed state, other thermodynamic properties of the system • Employed for the selection of green solvents, ionic liquids, etc. For the dissolution of lignin
Simulation models	Quantum chemical simulations	Investigates the underlying molecular interactions in the solute/solvent system	–	• Helps to predict the solvents’ ability to cause solute dissolution • Helps in the selection of ionic solvents for lignin extraction
	Molecular dynamics simulations	Provides snapshots of reaction mechanisms, studies the dynamic behavior of the molecular systems	–	• Helps to identify unique combinations of biomass–ionic solvent systems
Loss tangent (tan *δ*)	–	The ability of a solvent for the effective conversion of microwave into heat energy	The ratio of the dielectric loss factor to the dielectric constant of the solvents	• Solvents with high tan *δ* are known to perform well under microwave heating systems

*δ*_d_, Dispersive or non-polar interactions; *δ*_p_, polar interactions between permanent dipoles; *δ*_h_, hydrogen bonds; *R*_a_, the distance between the solute and solvent in *δ* plotted on a 3-D scale; *R*_0_, radius of the solubility sphere plotted with different solvents used in trial-and-error method

### Combined severity factors of organosolv

The severity factor [Log(*R*_0_)] is the blend of LCB pretreatment duration of time as well as the temperature (relative to the boiling point of pure water, *T*_ref_ = 100 °C) into a sole parameter. Combined severity factor (CSF) is a more usually used tactic that combines the reaction time, temperature, addition of catalysts, and pH into a single factor (Chum et al. 1990; Zhang et al. 2016b). These are very effective parameters in determining the pretreatment severity, effectiveness, etc. (Montané et al. 1998).

The severity factor is calculated, as shown in the following equation (Montané et al. 1998):

1 $${\text{Log}}\left( {R0} \right) = \frac{{{\text{Log}}\left( {t\exp \left( {T - T{\text{ref}}} \right)} \right)}}{14.7}.$$

The CSF is a modification of this equation with pH consideration as shown in the following equation (Chum et al. 1990):

2 $${\text{CSF}} = {\text{Log}} \left( {R0} \right) - {\text{pH}}{.}$$

Goh et al. (2011) used CSF to optimise the ethanol organosolv pretreatment for empty palm fruit bunch and concluded that xylan and lignin extraction could be predicted using CSF. A modified CSF was studied for the optimization of barley straw organosolv and was found that a logarithm range between 0.5 to 0.7 was most suitable for pretreatment. The pretreatment conditions placed within the range, with temperature 140 °C, 20 min reaction time, 35 mol/m^3^ sulphuric acid concentration (Log(*R*_0_) = 0.6) was found to result in a high xylose recovery of 66.7% and 75.4% enzymatic digestibility (Salapa et al. 2018). CSF varies with the LCB type and hence was also optimised between 1.0 to 2.5, for respectable recovery of cellulose as well as enhanced enzymatic digestibility. A higher CSF was better in terms of delignification and hemicellulose removal as compared to a low CSF value (Zhou et al. 2018).

Reactor configurations and the scale of the process also affect the correlation between the pretreatment severity and enzyme-assisted hydrolysis. A high-severity organosolv process is generally employed for hemicellulose removal and delignification. The pitfall of this process is the noteworthy cellulose degradation and sugars pleading to the production of enzyme/fermentation inhibitors (Vaidya et al. 2022). Jang et al. (2016) adjusted the pretreatment conditions for a CSF value of 1.16 in ethanol organosolv pretreatment of *Liriodendron tulipifera*. It was found that when CSF was kept constant, the pH value when increased from 0.72 to 1.90 decreased the lignin yield from 12.9% to 11.2%, even though the increased reaction temperature from 130 to 170 °C. This showed that in the case of pretreatment with similar severity factors, manipulation of individual pretreatment factors shall be employed to govern by-product properties while simultaneously keeping the efficiency for production of glucose.

### Solubility parameters of organosolv

The solubility of diverse polymers in organosolvents can be explained using the solubility parameter (*δ*) theory, useful for non-polar and slightly polar polymers (Sameni et al. 2017). The act of solvents in the dissolution of lignin determines the delignification efficiency in the organosolv process. Under this context, the solubility parameters help in understanding the underlying mechanisms regarding the lignin solubility in a particular organic solvent. The principle of solubility theories relies on the solubility between two liquids, and their intermolecular interactions between the first (A–A) and second (B–B) components which should be in a similar order of magnitude, and thus can be broken from A–B interactions (Novo and Curvelo 2019). Hildebrand established the foremost theory of solubility parameters, wherein the polymer solubility is described as the square root of the cohesive energy density (Eq. 3) ():

3 $${\updelta } = \sqrt {\frac{E}{{{\text{Vm}}}}}$$

where *E*/Vm is the cohesive energy density (cal/cm^3^), *E* is the cohesive energy (cal/mol) and Vm is the molar volume (cal/cm^3^).

Hildebrand solubility parameter was not universally accurate and does not accurately discriminate the intermolecular interactions. Thus, further modification in the solubility parameters by Hansen et al. (2007) was adapted for determining solubility parameters and was improved on three diverse kinds of molecular interactions; (i) polar interactions between permanent dipoles (*δ*_P_), (ii) Dispersive or non-polar interactions (*δ*_D_), and (iii) hydrogen bonds (*δ*_H_). Here, the square root of the total of the quadratics of the three interactions constitutes the solubility parameter, *δ* (Hansen et al. 2007; Novo and Curvelo 2019). The parameters can be plotted on a 3-D scale and from it, the “distance” (*R*_a_) among the solute and solvent can be calculated. The solvents tested by the trial-and-error system in the 3-D space can be plotted as a sphere (solubility sphere). The radius of the sphere is calculated as the “interaction radius”, *R*_0_. The relative energy difference (RED) is calculated as the ratio between *R*_a_ and *R*_0_ (Eq. 4):

4 $${\text{RED}} = \frac{{{\text{Ra}}}}{R0}.$$

It is assumed that solvents comprised within the inside of the sphere, or slightly on the surface area (RED ≤ 1), are considered good solvents for study. A RED value > 1 shows a low affinity of the solvent with the solute. This infers that *R*_a_ should have a value lesser than *R*_0_ (Hansen 2000; Sánchez-Camargo et al. 2019).

### COSMO-RS methods: understanding dissolution of organosolv mechanism

Another powerful approach employed for understanding the pathway of dissolution in liquid is called the Conductor-like Screening Model for Real Solvents (COSMOS). The arithmetic simplicity, numerical stability, and high insensitivity to outlying charge errors are the merits of this system. A further advanced model COSMO-RS (COSMO for Realistic Solvation) combines COSMOS with statistical thermodynamics for the estimation of thermodynamic properties deprived of using investigational data (Klamt 2017). Initially, the interest of the molecule is entrenched into a computer-generated conductor to produce polarization charge density on its external surface. Throughout the quantum chemical COSMO calculation, the molecule converges to its energetically optimal state. During the second step, the stored COSMOS results are used for a thermodynamic calculation to enumerate the energy of the pairwise interacting segments concerned with the molecular interaction manners. The *σ*-profile of the molecule is outlined from the histogram of the charge intensities around each molecule from the 3-D distribution of polarization charges. This explains the affinity of the selected compound for its interaction with the solvents in a mixed state as well as the other thermodynamic properties of the system (Aissou et al. 2017; Sánchez-Camargo et al. 2019). COSMOS-RS has been utilized for the selection of ionic liquids, green solvents, etc. for lignin dissolution (Balaji et al. 2012; Yu et al. 2022b).

### Quantum chemical (QC) and molecular dynamics (MD) simulation models: molecular mechanism insights

Simulation models are essentially used for understanding the mechanism of dissolution of LCB and its components. The Molecular Dynamics (MD) simulations and Quantum Chemical (QC) deliver fundamental understandings of the molecular classifications and structure of lignin, ionic liquid systems, etc. which can be manipulated for selecting organosolv systems. QC models give a deeper molecular mechanistic to understand the action of different solvents in dissolving the lignin. QC calculations are used for the investigation of underlying molecular interactions in the solute/solvent system. It helps to provide a better understanding of the drive of the solvent’s ability for solute dissolution (Achinivu et al. 2021). The QC models are very useful in the selection of ionic liquid solvents for lignin extraction. MD simulation snapshots of the mechanisms are also recorded for a deeper understanding of the different interactions in the systems (Ji and Lv 2020).

These computational techniques have also been used for the determination of limitless investigation of each unique combination of ionic liquid solvents and LCB. QC and MD models enable the understanding of the dominating factors that govern the efficiency of solvent-assisted LCB pretreatment. They give an idea about the interaction of different components of LCB with the pretreatment solvents. This helps in predicting the fractionation efficiency of a specific or a class of solvents used in the study. The MD simulations are utilized to study the dynamic behaviour of the molecular systems that help in understanding the interactions of solvent–biomass during the pretreatment system. In comparison, relative force fields simulate and explain in reactions detail the bond-breaking/forming. Another system using multiscale modeling has also helped in the investigation of reaction mechanisms, physical and chemical properties, and the overall dynamics of the system. These systems, when combined with machine learning interfaces have the potential to provide tools for the rapid evaluation of new solvents, deconstruction processes, etc. (Pham et al. 2022). De Santi et al. (2021) performed a computational study on the acid-assisted lignin β‑*O*‑4 linkage breakdown with stabilization of ethylene glycol via using ReaxFF molecular dynamics, where they setup a realistic scenario of a typical experiment using solute, catalyst, and reagent. The work led to the revelation of the H_2_SO_4_ role (as a proton donor) in the lignin β-*O*-4 acidolysis and their pathways for ethylene glycol stabilisations. The work strategy using the combined experimental and computational work was found to be efficient towards designing reactions and experiments.

### Loss tangent for solvent selection in microwave system

The loss tangent (tan *δ*) characterises have been used for the screening and selection of specific solvents for the effective conversion of microwave into heat energy. It is described as the dielectric loss factor to the dielectric constant ratio of the solvents. Based on the loss tangent value, the solvents are classified into low (tan *δ* < 0.1), medium (0.1 < tan *δ* < 0.5), and high (tan *δ* > 0.5) microwave absorbing solvents. It was experimentally found that the microwave absorbing tendency and polarity of the solvents deeply influences the distribution in the molecular weight of lignin (Dhar and Vinu 2017). Calculation of the loss tangent of the solvent in the study gives additional information on process efficiency when organosolv systems are combined with microwave systems. In a study, glycerol was selected for the pretreatment of softwood due to its high dielectric loss factor. It was observed that the microwave–glycerol system gave a high saccharification yield (Liu et al. 2010). However, it is also to be noted that the solvents that display a low tan *δ* value may need not be inevitably eliminated from microwave systems as long as high-intensity electric fields are supported in the system (Kostas et al. 2017).

The dielectric constant and the dielectric loss factor were found to increase with temperature for microwave-pretreated samples. An upsurge in temperature causes a rise in the ionic conductivity, thereby increasing the dielectric constant and loss factor (Gaber et al. 2020). The add-on of H_2_O in the system leads to increased conductivity of the solvent, the dielectric constant and the dielectric loss factor might also improve with it (Isci et al. 2021). Various solvents with high tan *δ* values are yet to be discovered with microwave assistance for LCB pretreatment. Some solvents that are useful for microwave-assisted organosolv process with this respect with high loss tangent, low cost, and ready availability include isopropyl alcohol, ethylene glycol, methanol, formaldehyde, etc. (Amini et al. 2021).

## Caveats and pitfalls of organosolv methods

Though the organosolv method is highly efficient as a pretreatment process, some demerits shadow the efficiency of the process. One of the major pitfalls of using organic solvents lies in lignin recovery. It is an energy-demanding, time-consuming tactic and uses solvents that are toxic to humans. Centrifugation has been found to be effective for lignin recovery, but the overbearing cost of these systems is a major obstacle in their commercialization. In other cases, the high viscosity of the solvent also affects the proper filtering of lignin. Precipitation methods using acidified water, air floatation, etc. are some of the other methods used for lignin recovery. From the ecological and economical perspective, it is suggested that the process must avoid the additional use of chemicals and move forward to using technologies, such as water precipitation (Ramírez-wong et al. 2014). Though the organosolv technology is highly effective in obtaining by-products in their pure forms which can further be processed into high-value biochemicals, the high temperature and pressure requirements of the desired process add-on to the drawback of the system. In addition, it is noted that the use of mineral acid catalysts in the systems raises environmental concerns as well as corrodes the systems/instruments of use. Thus, using organic acids in the process is very hard to handle. Moreover, lignin recovery requires expensive organic solvents, efficient control systems, high energy requirements, etc. (Bensah and Mensah 2013).

Moreover, the flammability of solvents also points to their safety risks. Solvents such as methanol are highly viscous and have a low boiling point, generating inflammable vapours at even low temperatures. Besides, ethanol requires high-pressure conditions to reduce the safety risks for the organosolv process, due to its high volatility. Similarly, other solvents are also known to have the risk of explosion due to their volatile nature, so the process requires extreme attention and control (Borand and Karaosmanoǧlu 2018). High flames of harsh solvents, huge fire explosions, etc. may occur unless proper safety measures have been taken (Tayyab et al. 2018). The high pressure and temperature conditions of the solvents consume a lot of power and higher investment will be required for the same (Maurya et al. 2015; Khaw et al. 2017). This will further upsurge the operational expenditure of the process. To reduce energy consumption, radiation-assisted heating methods, e.g., microwave energy, could be used which has been shown to reduce the reaction time considerably (Alio et al. 2019). Energy consumption is also increased during the solvent recovery process (Borand and Karaosmanoǧlu 2018). The removal of solvents from the system is also very important as they are also known to inhibit enzymatic hydrolysis, fermentative microorganisms, etc. (Xu and Huang 2014).

## Life cycle assessment and techno-economic assessment of organosolv system

The LCA and TEA of various organosolv systems were studied by scientists to record the environmental and economic viability of the process. Bello et al., (2018) conducted LCA analysis on residual beech woodchips using organosolv fractionation. The study concluded that the hotspots of the system include the pretreatment of LCB, energy demands of the system as well as enzyme production. It was observed through eco-efficiency indicator analysis that integration of the multi-production system or biorefinery would provide improved results as the processing steps and production volume fit the environmental and techno-economic necessities. Another LCA done on the integral revalorization of vine shoots showed that the scenarios incorporating organosolv delignification or fermentation of glycerol liquors showed the worst environmental profiles. The higher energy and chemical requirements were a major drawback from the environmental perspective (Gullón et al. 2018). Laure et al. (2014) reported that the emissions by the organosolv LCB biorefinery were 50–80% lower as compared to the reference methods. However, a major obstacle of the desired process is the increment of forest land utilization in comparison with fossil feedstock refineries (Prasad et al. 2016).

In organosolv biorefinery systems which completely utilize LCB (formic acid pretreatment), it was observed that the system was superior in environmental performance, excluding the ozone layer depletion. An expanded supply chain of the process showed positive results in GHG reduction and was also concluded as a profitable system (Liu et al. 2021). The LCA study of different LCB pretreatment methods by Prasad et al. (2016) concluded that the organosolv process in general was good in terms of environmental aspects, but the high emission of CO_2_ by the process during bioethanol production was a major setback. However, compared to other traditional systems, it was observed that the CO_2_ and SO_2_ emissions were lower in this case. In addition, from the economical perspective, an organosolv-assisted biorefinery generates both bio-based ethanol and natural chemicals compared to processes that only produce bioethanol (Zhang et al. 2016a). A valorisation study of almond shells shows that the organosolv process had major environmental issues due to abiotic depletion, photochemical oxidation, etc. (Sillero et al. 2021). However, at the same time, WB fractionation using the organosolv method has been recognized as the most environmentally friendly tactics/approaches for the generations of biochemical products (Ryan and Yaseneva 2021).

TEA analysis of an integrated biorefinery using the organosolv pretreatment was found to be profitable with a condition of a long payback period of 16.8 years (Bulkan et al. 2021). To progress in the economic efficiency of the organosolv-assisted process, the solvent needs to be constantly recovered and recycled back. However, the inhibition products formed during LCB fractionation become a major drawback of the process (da Silva et al. 2018a). Organosolv systems with higher energy requirements also increase the cost of the process. However, as the process uses less water, enables better usage of LCB and savings in equipment costs; the process holds an upper hand in preference over other pretreatment methods. Efficient lignin removal, a higher amount of ethanol production, etc. increases the probability of savings potential during the downstream purification processes. Additional developments in the process are predicted to minimize the usage of solvent, elevated solid loading, and upsurge the ethanol ratio in the organosolv, which direct towards a savings of 43.3% in minimum ethanol selling price as compared to other methods (da Silva et al. 2018b). Kabir et al. (2015) testified that out of different organic solvents used in their study, i.e., methanol, ethanol, and acetic acid. Among them, acetic acid and ethanol pretreatment led to higher methane yields. However, the TEA of the process showed that methanol pretreatment was economically more viable than the others. This is because the methanol recovery is inexpensive compared to the other organosolvents used in the study, which led it to be a profitable process. TEA of a two-step organosolv pretreatment and enzyme-assisted hydrolysis on sugarcane bagasse for generation of bioethanol production. Mesa et al. (2016) proposed an alternative double-step organosolv method with high economic benefits. The first step included a pulping acid solution (absence of ethanol) for 15 min, followed by a second step utilizing 45% (v/v) ethanol for 60 min and NaOH (3%) on dry fiber. The above-experimented pretreatment technology was found to have improved yields of ethanol with low OPEX and hence might be established on an industrial level. In addition, the high-quality lignin obtained during this process is initially unaltered and little condensed in comparison with kraft lignin. This lignin can be further processed as a green polymer for the production of biopolymers which adds to the financial stability and environmental advantages of organosolv-assisted systems.

## Conclusion

The organosolv method is an excellent pretreatment strategy to generate renewable biofuel, biomaterials, and other valuable product synthesis. The LCB processing can be enhanced via organosolv pretreatment along with the integration of the latest technological advancements. The mechanism of action of the organosolv method mainly involves the aryl ether bond breakage between the LCB components, which can be improved by the add-on of novel catalysts. Further to improve the organosolv pretreatment various aspects such as combined parameters, solubility parameters, etc. should additionally be considered. Finally, the caveats and pitfalls have to be carefully analysed for high yield end product generation along with economic viability and ultimately contributing sustainably towards the welfare of society and the ecosystem.

## Data Availability

Data sharing is not applicable as no data sets were generated or analysed in the review. All other materials used in the study are duly cited in the review.

## Abbreviations

LCB: Lignocellulosic biomass
GHG: Greenhouse gas
HSP: Hansen solubility parameters
COSMO-RS: Conductor-like Screening MOdel for Real Solvents
QC: Quantum chemical
MD: Molecular dynamics
3-D: Three-dimensional
CSF: Combined severity factor
LBPA: Low boiling point alcohol
HBPA: High boiling point alcohols
MIBK: Methyl isobutyl ketone
SSF: Simultaneous saccharification and fermentation
THF: Tetrahydrofuran
WB: Woody biomass
2-MeTHF: 2-Methyl tetrahydrofuran
GVL: γ-Valerolactone
FTIR: Fourier-transform infrared spectroscopy
SEM: Scanning electron microscopy
NMR: Nuclear magnetic resonance
XRD: X-ray diffraction
EAOP: Electro-assisted organosolv pretreatment
3H2NA: 3-Hydroxy-2-naphthoic acid
TBHQ: Tert-butylhydroquinone
BHA: 3-Tert-butyl-4-hydroxyanisole
BHT: Butylated hydroxytoluene
RED: Relative energy difference
tan *δ*: Loss tangent
LCA: Life cycle assessment
TEA: Techno-economic assessment
